# SELF-MANAGEMENT SUPPORT STRATEGIES BASED ON THE RNAO GUIDELINES IN AECOPD PATIENTS: A RANDOMISED CONTROLLED TRIAL

**DOI:** 10.1093/geroni/igad104.3786

**Published:** 2023-12-21

**Authors:** Weisi Peng, Weixiang Luo, Xiufen Yang, Chaoran Qu, Di Huang, Yan Liu, Yi Fan, Weiwei Zhang

**Affiliations:** Shenzhen People’s Hospital, Shenzhen, Guangdong, China (People’s Republic); Shenzhen People’s Hospital, Shenzhen, Guangdong, China (People’s Republic); Shenzhen People’s Hospital, Shenzhen, Guangdong, China (People’s Republic); Shenzhen People’s Hospital, Shenzhen, Guangdong, China (People’s Republic); Shenzhen People’s Hospital, Shenzhen, Guangdong, China (People’s Republic); Shenzhen People’s Hospital, Shenzhen, Guangdong, China (People’s Republic); Shenzhen People’s Hospital, Shenzhen, Guangdong, China (People’s Republic); Shenzhen People’s Hospital, Shenzhen, Guangdong, China (People’s Republic)

## Abstract

Abstract Chronic disease management is a key intervention for the management of AECOPD patients. We conducted a Chinese-based trial according to RNAO’s chronic disease management guidelines.The aim of this study was topply an evidence-based guideline practice protocol for self-management in patients with chronic disease to improve the clinical outcomes, as well as reduce the economic costs for patients discharged with AECOPD.In this study, the “Strategies to Support Self-Management in Chronic Conditions: Collaboration with Clients”, published by RNAO, was used as a practice guideline to be applied in the follow-up of patients discharged from COPD to understand patients’ post-discharge self-management ability, pulmonary function, and readmission within one year, and to compare the cost-effectiveness for patients after discharge. The follow-up of both groups was conducted over time, and the patients’ self-management ability and pulmonary function in the observation group were effectively improved at month 6 and month 12 and was significantly different in comparison with the control group (P< 0.05). The observation group showed significant differences in readmission rates and economic costs at month 12 as compared to the control group (P< 0.05).Application of the guideline’s chronic disease self-management support strategies could stabilize patients’ blood oxygenation levels, better maintain pulmonary function, significantly improve patients’ self-management ability, and reduce patients’ medication costs. Keywords: COPD;Chronic disease self-management; RCT; economic analysis

